# Mesenchymal Stem Cells from Human Extra Ocular Muscle Harbor Neuroectodermal Differentiation Potential

**DOI:** 10.1371/journal.pone.0156697

**Published:** 2016-06-01

**Authors:** Darilang Mawrie, Atul Kumar, Damaris Magdalene, Jina Bhattacharyya, Bithiah Grace Jaganathan

**Affiliations:** 1 Stem Cell and Cancer Biology Lab, Department of Biosciences and Bioengineering, Indian Institute of Technology Guwahati, Guwahati, Assam, India; 2 Department of Strabismus, Sri Sankaradeva Nethralaya Hospital, Guwahati, Assam, India; 3 Department of Hematology, Gauhati Medical College and Hospital, Guwahati, Assam, India; University of Torino, ITALY

## Abstract

Mesenchymal stem cells (MSC) have been proposed as suitable candidates for cell therapy for neurological disorderssince they exhibit good neuronal differentiation capacity. However, for better therapeutic outcomes, it is necessary to isolate MSC from a suitable tissue sourcethat posses high neuronal differentiation. In this context, we isolated MSC from extra ocular muscle (EOM) tissue and tested the *in vitro* neuronal differentiation potential. In the current study, EOM tissue derived MSC were characterized and compared with bone marrow derived MSC. We found that EOM derived MSC proliferated as a monolayer and showed similarities in morphology, growth properties and cell surface marker expression with bone marrow derived MSC and expressed high levels of NES, OCT4, NANOG and SOX2 in its undifferentiated state. They also expressed embryonic cell surface marker SSEA4 and their intracellular mitochondrial distribution pattern was similar to that of multipotent stem cells. Although EOM derived MSC differentiated readily into adipocytes, osteocytes and chondrocytes, they differentiated more efficiently into neuroectodermal cells. The differentiation into neuroectodermal cellswas confirmed by the expression of neuronal markers NGFR and MAP2B. Thus, EOM derived MSC might be good candidates for stem cell based therapies for treating neurodegenerative diseases.

## Introduction

Adult stem cells are used extensively for tissue regeneration, repair and also used successfully in several instances to correct genetic disorders in patients [1–4]. In addition to detailed characterization of the nature of these adult stem cells, there is also a need to identify novel tissue sources from where stem cells could be isolated and manipulated for therapeutic purposes. Adult stem cells from different sources do not differentiate equally into all lineages unlike embryonic stem cells [5]. The differentiation potential of adult stem cells have been closely related to their tissue of origin [6] eventhough they could be induced to trans-differentiate into cells of different germ layer in the presence of induction factors. Mesenchymal stem cells from bone marrow, adipose tissue and umbilical cord blood could differentiate into several mesenchymal as well as non-mesenchymal lineage cell types [7]. These cells have been converted into adipogenic, osteogenic and chondrogenic lineage cells with relatively high efficiency and they functioned and repaired effectively *in vivo* as well [7]. One of the major areas where cell therapy is much sought after is neuronal repair for spinal cord injury and neurodegenerative diseases. One of the drawbacks associated with using embryonic or tissue specific adult stem cells for neuronal repair is its conversion into cells of redundant lineages *in vivo*. However, adult multipotent cells which could rapidly differentiate into neuronal cells but show reduced differentiation into other cell types would be an advantage in this scenario.

In the current study, stem cells were isolated from extra ocular muscle tissue and their multipotent differentiation ability was analyzed. Extra ocular muscles (EOM) are a group of highly specialized muscle type that control the eye movements and are distinct from the other skeletal muscles [8] and possess a unique quality of being spared in muscular dystrophies [9, 10]. EOM tissue is excised out during corrective surgery for strabismus when a resection (strengthening) is performed. Recent evidences suggest that EOM possesses a high number of stem cells compared to the limb muscles and have highproliferative capacity [11]. The stem cell compartment of EOM cells were determined by their ability to exclude the dye Hoechst 33342 to give rise to side-population (SP) cells [12]. EOM derived SP cells alsoshowed high hematopoietic regenerative capacity after *in vivo* transplantation [13].

We hypothesized that since EOM tissue is distinct from other tissue types, and highly innervated unlike skeletal muscle, these cells might posses a superior neuronal differentiation capacity. To test this hypothesis, we first studied the growth, differentiation potential and gene expression profiles of EOM derived stem cells and compared them with the bone marrow derived MSC which have multi-lineage differentiation capacity. In the current study, for the first time, we identified MSC from EOM tissue that shared gene expression and phenotype profiles with bone marrow derived MSC. They also differentiated into mesodermal, neuroectodermal cells and indicate a novel source of cells for regenerative therapy.

## Materials and Methods

The current study was reviewed and approved by Institute Human Ethics Committee (IHEC) of Indian Institute of Technology Guwahati (IITG).

### Chemicals and Reagents

Dulbecco’s modified eagle’s medium (DMEM), fibronectin, leukocyte alkaline phosphatase kit, Oil red O, Safranin O, dexamethasone, iso butyl methyl xanthine, indomethacin, insulin, β- glycerophosphate and ascorbic acid were purchased from Sigma Aldrich (Steinheim, Germany). Tissue culture plastic plates and flasks were from BD biosciences (Heidelberg, Germany). Fluorescent conjugated anti-human antibodies were from BD biosciences. Anti-Oct4 antibody was from Santa Cruz. Fetal bovine serum (FBS), recombinant human BDNF, chondrogenic differentiation media, neurobasal media, neuronal supplements and Tetramethylrhodamine, ethyl ester (TMRE) were purchased from Thermofisher scientific (Paisley, UK).

### Extra Ocular Muscle Tissue Collection

EOM samples were obtained from patients undergoing corrective surgery for strabismus in collaboration with the Department of Pediatric Ophthalmology and Strabismus at Sri Sankaradeva Nethralaya Hospital after written informed consent and in accordance with the hospital human ethics committee guidelines. The tissues were collected in vials containing DMEM with antibiotics and processed within 12 hours. The tissue was rinsed briefly in PBS containing 2x antibiotics, mechanically dissociated with forceps and plated in DMEM containing 10% FBS. Fresh media was added regularly until colonies with spindle shaped cells were obtained.

### Bone Marrow Mesenchymal Stem Cells

Bone marrow samples were obtained from patients referred to Hematology department of Gauhati Medical College Hospital (GMCH) after written informed consent following GMCH human ethical committee guidelines. Bone marrow cells after red cell lysis were cultured in DMEM supplemented with 10% fetal bovine serum (FBS) at a cell density of 1x10^5^ cells/cm^2^. Complete media change was performed after 48 hours to remove the non-adherent cells and spindle shaped adherent colonies appeared after 2–3 weeks in culture.

### Field Emission Scanning Electron Microscope (FESEM) Analysis

Cells were grown on fibronectin coated coverslips, fixed with ice-cold acetone:methanol (1:1) solution and dehydrated with graded series of ethanol (50%, 70%, 90% and 100%). The cells were gold coated with a sputter coater and viewed under Field Emission Scanning Electron Microscope (Zeiss, Germany).

### Immunocytochemical Staining

Cells were washed with PBS and fixed with 4% paraformaldehyde for 20 minutes at room temperature. The cells were permeabilised with 0.1% triton X-100 for 20 minutes, washed and stained with primary antibody in 2% FBS solution at 4°C overnight and with fluorescently conjugated secondary antibody for 1-2hr at room temperature. Nucleus was stained with 4',6-Diamidino-2-Phenylindole, Dihydrochloride (DAPI, 5mg/ml) and cells were washed, mounted and documented microscopically (Zeiss, Axio Observer Z1).

### Phenotyping by Flow Cytometry

Adherent cells were trypsinized and stained with fluorescent dye conjugated monoclonal antibodies against human cell surface antigens (BD Biosciences). The cells were incubated at 4°C for 30 minutes, washed and analyzed with FACS calibur (Becton Dickinson). Propidium iodide was added before analysis for live/dead separation.

For intracellular markers, the cells were trypsinized and fixed with paraformaldehyde (4%) and permeabilised with TritonX-100 (0.1%) and stained with fluorescently conjugated antibodies for 1hr at room temperature.

### SSEA4 Staining

The cells were cultured on fibronectin coated cover slips for 24–48 hours. The cells were washed with PBS and incubated with anti-SSEA4 (Chemicon) antibody for 1 hour. The cells were washed and stained with fluorescent conjugated secondary antibody. The stained cells were microscopically documented.

### Colony Forming Unit Assay

One hundred cells per well were seeded in a 6-well plate and cultured in media containing 20% FBS for 14 days. The cells were fixed with ice-cold methanol and stained with May-Grünwald and Giemsa solution. Colonies with more than 50 cells were counted.

### Adipogenic, Osteogenic and Chondrogenic Differentiation

EOM-MSC and BM-MSC were differentiated into adipocytes and osteocytes using specific induction media as reported earlier [14]. Briefly, the cells were cultured in DMEM containing 10% serum supplemented with dexamethasone, isobutylmethylxanthine, indomethacin and insulin for adipogenic differentiation and dexamethasone, β-glycerophosphate, and ascorbic acid for osteogenic differentiation. After 21–28 days, differentiation was analyzed by staining for alkaline phosphatase, alizarin red to detect osteogenic differentiation and oil red-O for analysis of adipogenic differentiation. Expression of OSTEOCALCIN and ADIPOQ in osteo- and adipo-differentiated cells respectively were analyzed by real-time quantitative PCR.

For chondrogenic differentiation, micromass cultures were generated in a 12-well plate according to the manufacturer’s instructions (Thermofisher scientific). Media change was performed every three days and the differentiated cells were stained with Safranin O after 21 days. Expression of SOX9 was analysed in chondrogenic differentiated cells by real-time PCR.

### Neuronal Differentiation

The cells were plated in poly-l-lysine coated plates at a density of 1.5-2x10^5^ cells/cm^2^. For neuronal differentiation, culture medium was replaced with pre-induction medium composed of DMEM, FBS (20%), FGF2 (10 ng/ml), B27 (2%), Forskolin (50 μM), and 3-isobutyl-1-methylxanthine (IBMX, 250 μM) with 2-mercaptoethanol (2-ME, 100μM) and incubated for 24 h. To initiate neuronal differentiation, the pre-induction medium was removed, washed with PBS, and replaced with neuronal induction medium containing DMEM, FBS (2%), FGF2 (10 ng/ml), insulin—transferrin—selenium supplements (1%), B27 (2%), Forskolin (5 μM), IBMX (125 μM), BDNF (50 ng/ml) with 2-ME (100 μM), and all-trans retinoic acid (RA, 0.5 μM). The cells were incubated for an additional 14–21 days.

### Real Time Quantitative PCR

Total cellular RNA was isolated from EOM-MSC and BM-MSC using TriZol reagent (Invitrogen) according to manufacturer’s instructions. cDNA was synthesized by reverse transcription using MultiScribe reverse transcriptase (Applied Biosystems, Foster City, CA, USA) and Oligo dT primers (Sigma) at 37°C for 120 minutes. The conditions for the real-time polymerase chain reaction (PCR) were as follows: initial denaturation at 94°C for 10 minutes followed by 40 cycles of denaturation at 94°C at 15 seconds, primer annealing and extension at 60°C for 45 seconds and a final extension at 72°C for 10 minutes using Power SyBr Green real-time PCR master mix (Applied Biosystems) using 7500 Real-time PCR System (Applied Biosystems). The primers used were GAPDH forward 5'-GGGAAGGTGAAGGTCGGAGT-3', GAPDH reverse 5'-GGGTCATTGATGGCAACAATA-3', NESTIN forward 5’-TGGCTCAGAGGAAGAGTCTGA-3’, NESTIN reverse 5’-TCCCCCATTCACATGCTGTGA-3’, OCT4 forward 5’-CGTGAAGCTGGAGAAGGAGA-3’, OCT4 reverse 5’-CTCAAAGCGGCAGATGGT-3’, NANOG forward 5’-CAAAGGCAAACAACCCACTT-3’, NANOG reverse 5’-TCTGGAACCAGGTCTTCACC-3’, SOX2 forward 5’-GGAGCTTTGCAGGAAGTTTG-3’, and SOX2 reverse 5’-GCAAGAAGCCTCTCCTTGAA-3’. The gene expression levels in each sample were normalized to their respective GAPDH expression levels. The expression levels were quantified using ΔΔCt method.

### Clonal Cell Line Derivation

Clonal cell lines were derived from EOM-MSC through limited dilution by plating one cell per well in a 96 well plate. Presence of one cell/well was confirmed microscopically after few hours and proliferating clones were expanded further.

### Mitochondrial Staining

Mitochondria in EOM-MSC were visualized by staining with Tetramethylrhodamine, ethyl ester(TMRE) as described earlier [15]. Briefly, TMRE (100nM) was added to the cells in the culture media and incubated at 37°C for 30 minutes. Stained cells were visualized using Zeiss Axio Observer Z1 inverted microscope (Zeiss, Gottingen, Germany).

### Karyotype Analysis

For karyotype analysis, colchicine (0.1μg/ml) was added to the cells in culture for 16 hours. The cells were trypsinized and resuspended in 0.56% KCl solution for 30 minutes at 37°C. The cells were washed twice with glacial acetic acid and methanol mixture (1:3) and dropped into clean wet slides. The slides were dried, chromosomes were stained with Giemsa and documented.

### Data Analysis

Flow cytometry data was analysed using FlowJo software (Tree Star Inc). Karyotype figures were analysed using Image J software. Statistical analysis was performed with SPSS software and values of p<0.05 were considered statistically significant. The difference in gene expression between EOM-MSC and BM-MSC was analysed by Mann-Whitney non-parametric variables test. The difference in gene expression between control and osteogenic, adipogenic, chondrogenic and neuronal differentiated EOM-MSC samples were tested using paired samples t-test.

## Results

### Isolation, Cell Morphology and Growth Properties

**Fig 1:**
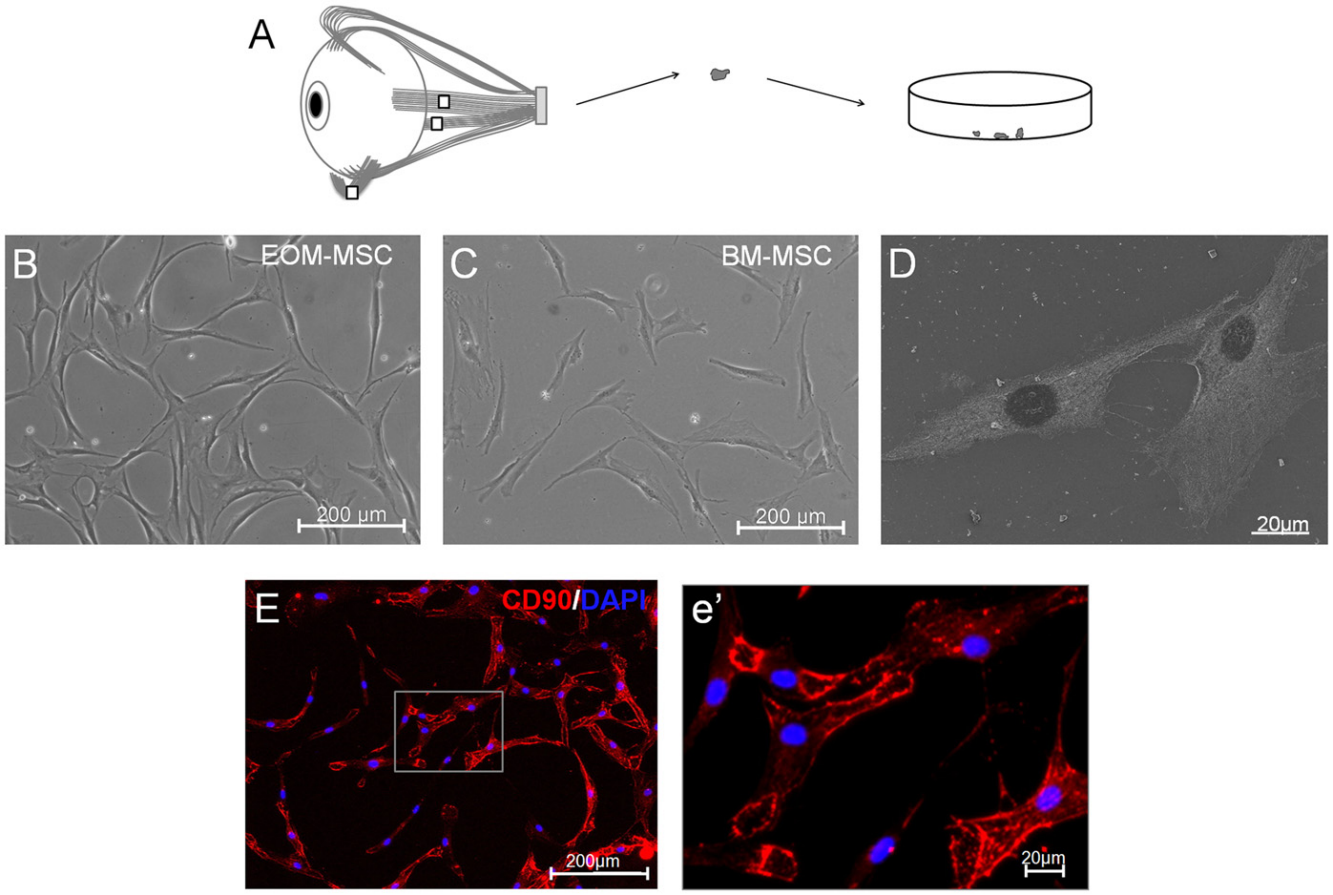
Isolation and expansion of EOM-MSC. (A) EOM-MSC were isolated from freshly resected EOM tissue from the human eye. The tissue was mechanically dissociated and plated in growth media. Phase-contrast microscopic images of (B) EOM-MSC and (C) BM-MSCshowing spindle shaped adherent cells, passage 2. (D) FESEM analysis of *in vitro* expanded EOM-MSC (passage 3). (E, e’) CD90 expression in *in vitro* expanded EOM-MSC determined by immunocytochemical staining. Representative photomicrographs are shown. EOM-MSC were from passage 2–4.

Fresh tissue samples of EOM resected from the human eye during corrective strabismus surgery of both male and female patients with a median age of 21 yrs were used in this study. The extra ocular muscle tissues obtained from the patients were of lateral rectus, medial rectus or inferior oblique muscle (Fig 1A). EOM tissues were mechanically dissociated and placed on fibronectin coated dishes and cultured in DMEM containing 10% fetal bovine serum. Within 2 weeks of plating, adherent spindle shaped cells emerged from the tissue and formed fibroblast-like colonies. These cells were harvested and further sub-cultured when they reached 70–80% confluency and were termed as EOM derived mesenchymal stem cells (EOM-MSC). EOM-MSC could be cultured *in vitro* for several passages without change in morphology, growth properties and karyotype. In the current study, the properties of EOM-MSC were compared with bone marrow derived MSC (BM-MSC) obtained from unrelated bone marrow donors.

Morphologically, the EOM-MSC closely resembled BM-MSC, forming spindle shaped cells and proliferated rapidly (Fig 1B–1D). EOM-MSC showed an average doubling time of 34 hours in culture whereas BM-MSC had a doubling time of 42 hours. By immunocytochemistry analysis of cultured cells, we found that EOM-MSC expressed CD90 and the expression did not vary significantly between the different donors (Fig 1E).

**Fig 2:**
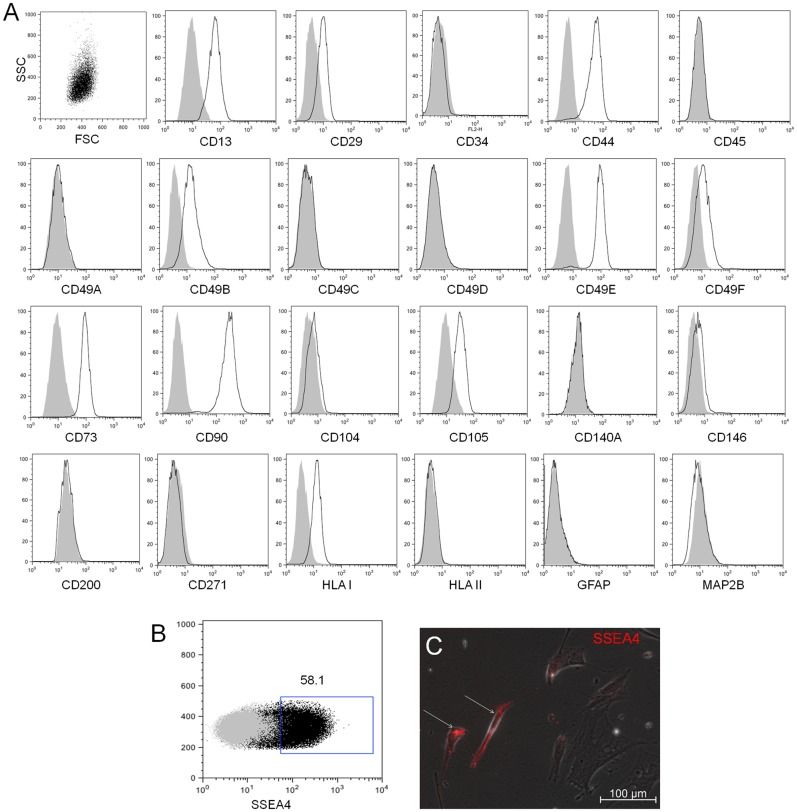
EOM derived cells express markers for MSC. (A) Representative histograms of flow cytometric analysis of EOM-MSC showing cell surface expression for CD13, CD29, CD34, CD44, CD45, CD49A, CD49B, CD49C, CD49D, CD49E, CD49F, CD73, CD90, CD104, CD105, CD140a, CD146, CD200, CD271, HLA I, HLA II, GFAP and MAP2B. Grey histogram represents the isotype control and black line represents the stained sample. EOM-MSC were at passage 2–5. (B) Dot plot showing the flow cytometric analysis of SSEA4 (stage specific embryonic antigen 4) expression in EOM-MSC and (C) fluorescent microscopic picture of EOM-MSC stained with antibody against SSEA4. The arrows indicate spindle shaped cells stained positive for SSEA4. Representative microscopic images are shown, EOM-MSC were from passage 3.

To analyse EOM-MSC further, the cell surface antigen expression profile was investigatedusing flow cytometry analysis. EOM-MSC from different donors expressed several MSC markers such as CD13 (92.37%±16.34), CD29 (21.44%±20.93), CD44 (68.89%±24.79), CD49B (48.93%±24.73), CD49E (73.25%±24.81), CD73 (73.90%±27.73), CD90 (97.4%±2.96), CD105 (70.17%±26.23), HLA Class I (78%±27.16) but did not express hematopoietic marker CD45, muscle stem cell marker CD34, neuronal markers CD271 (NGFR), GFAP, MAP2B and CD104, CD140Aor HLA class II (Fig 2A). The expression levels of cell surface markers in EOM-MSC was comparable to that of BM-MSC, except CD49B which was expressed significantly higher in EOM-MSC compared to BM-MSC (48.93%±24.73% in EOM-MSC versus 1.37%±0.44% in BM-MSC, p = 0.0285). We also identified cell surface expression of stage specific embryonic antigen 4 (SSEA4; 57.3%±2.98) by flow cytometry (Fig 2B). Byimmunocytochemistry, it was observed that SSEA4 was expressed predominantly in the spindle shaped cells (Fig 2C). The cell surface marker expression profile of EOM-MSC did not change significantly between different donors or over different passages (passage 2–8).

### Differentiation of EOM-MSC into Mesodermal Lineage

**Fig 3:**
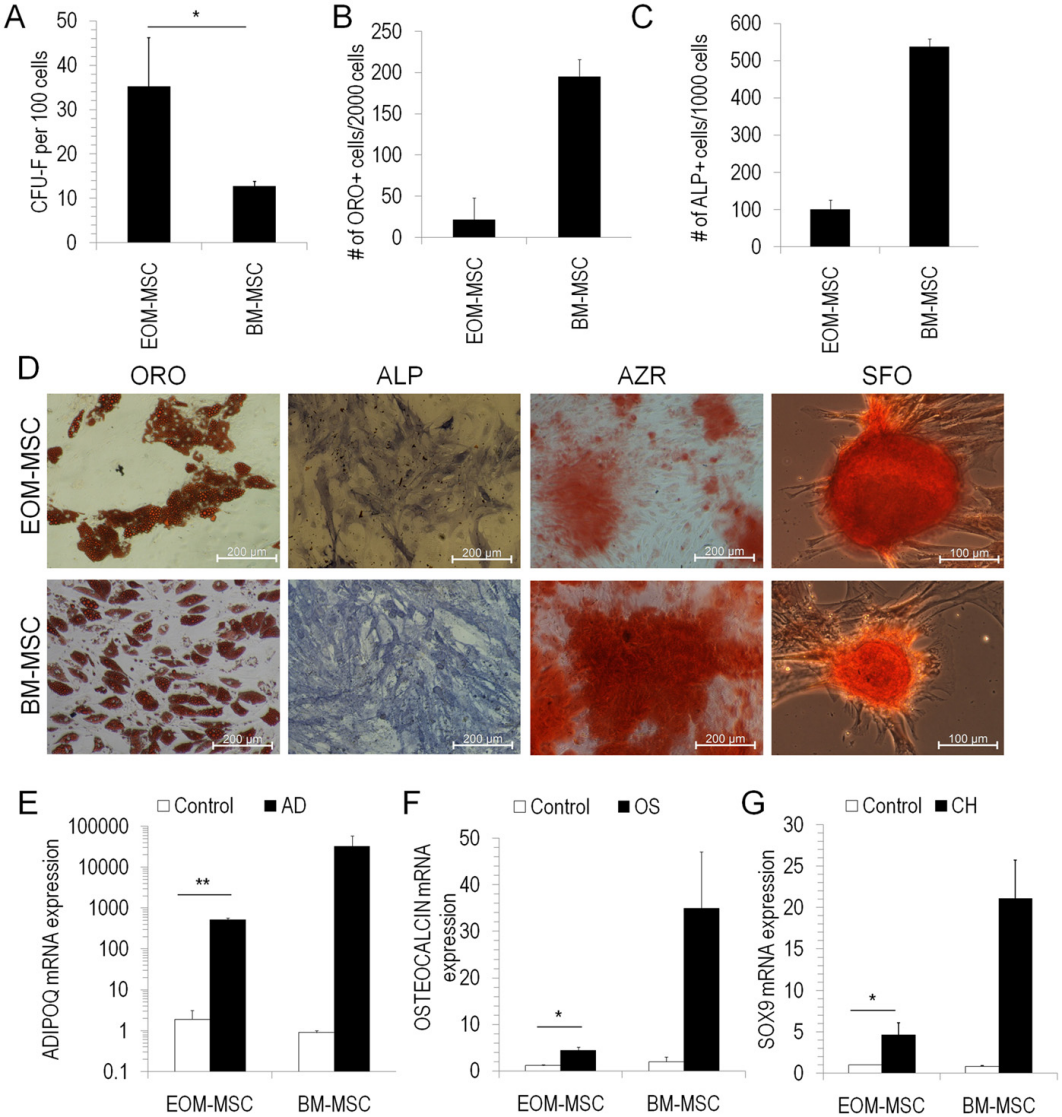
CFU-F ability and mesodermal differentiation of EOM-MSC. (A) CFU-F (colony forming unit-fibroblasts) assay was performed to analyse the colony forming ability of EOM-MSC and BM-MSC. Mean±SEM, n = 6–12, *p<0.05. The cells were from passage 3–4. (B-G) EOM-MSC and BM-MSC were differentiated into adipocytes, osteocytes or chondrocytes for 21–28 days. (B, D) Adipogenic differentiation was determined by staining with oil red O, (C, D) osteogenic differentiation by staining with alkaline phosphatase and alizarin red and (D) chondrogenic differentiation was determined by safranin O staining. The number of oil red O (ORO) positive cells per 2000 cells and alkaline phosphatase (ALP) positive cells per 1000 cells were calculated for EOM-MSC and BM-MSC after differentiation into respective lineages. Values are Mean±SEM, n = 4–7. (E-G) Real-time PCR was performed to determine the expression levels of ADIPOQ, OSTEOCALCINor SOX9 in undifferentiated (CONTROL), adipo-differentiated (AD), osteo-differentiated (OS) or chondro-differentiated (CH) EOM-MSC and BM-MSC. Values are Mean±SEM, n = 3. The expression levels were normalized to GAPDH expression levels in the respective samples. EOM-MSC and BM-MSC were from passages 3–5 *p<0.05. ORO-Oil red O, ALP-alkaline phosphatase, AZR-Alizarin red, SFO-Safranin O.

The ability to form a single-cell derived colony or the CFU-F (colony-forming unit-fibroblasts) ability of EOM-MSC was analysed and compared with BM-MSC. The CFU-F ability of EOM-MSC was significantly higher than that of BM-MSC (35.17%±11.05% in EOM-MSC versus 12.76%±1.10% in BM-MSC) implying the high proliferation capacity of EOM-MSC (Fig 3A). Since EOM-MSC resembled BM-MSC closely in their morphology, phenotype and growth properties, their differentiation ability into adipocytes, osteocytes and chondrocytes was tested. EOM-MSC at early passage was induced to differentiate into adipocytes, osteocytes or chondrocytes by addition of specific induction factors and the differentiation percentage was determined. EOM-MSC readily differentiated into adipocytes, osteocytes and chondrocytes as determined by specific staining to detect the differentiation (Fig 3B–3D) and control uninduced cells did not stain for the any of the differentiation markers (data not shown). Differentiation into adipocytes, osteocytes and chondrocytes was further confirmed by gene expression analysis and we observed a significant upregulation of mRNA levels of *ADIPOQ* in adipocyte differentiated cells, *OSTEOCALCIN* in cells differentiated into osteocytes and *SOX9* in chondrogenic differentiated cells (Fig 3E–3G). However, we noticed that the differentiation ability of EOM-MSC into either adipocytes, osteocytes or chondrocytes waslower than that seen in BM-MSC (Fig 3B–3G).

### Differentiation of EOM-MSC into Neuroectodermal Lineage

**Fig 4:**
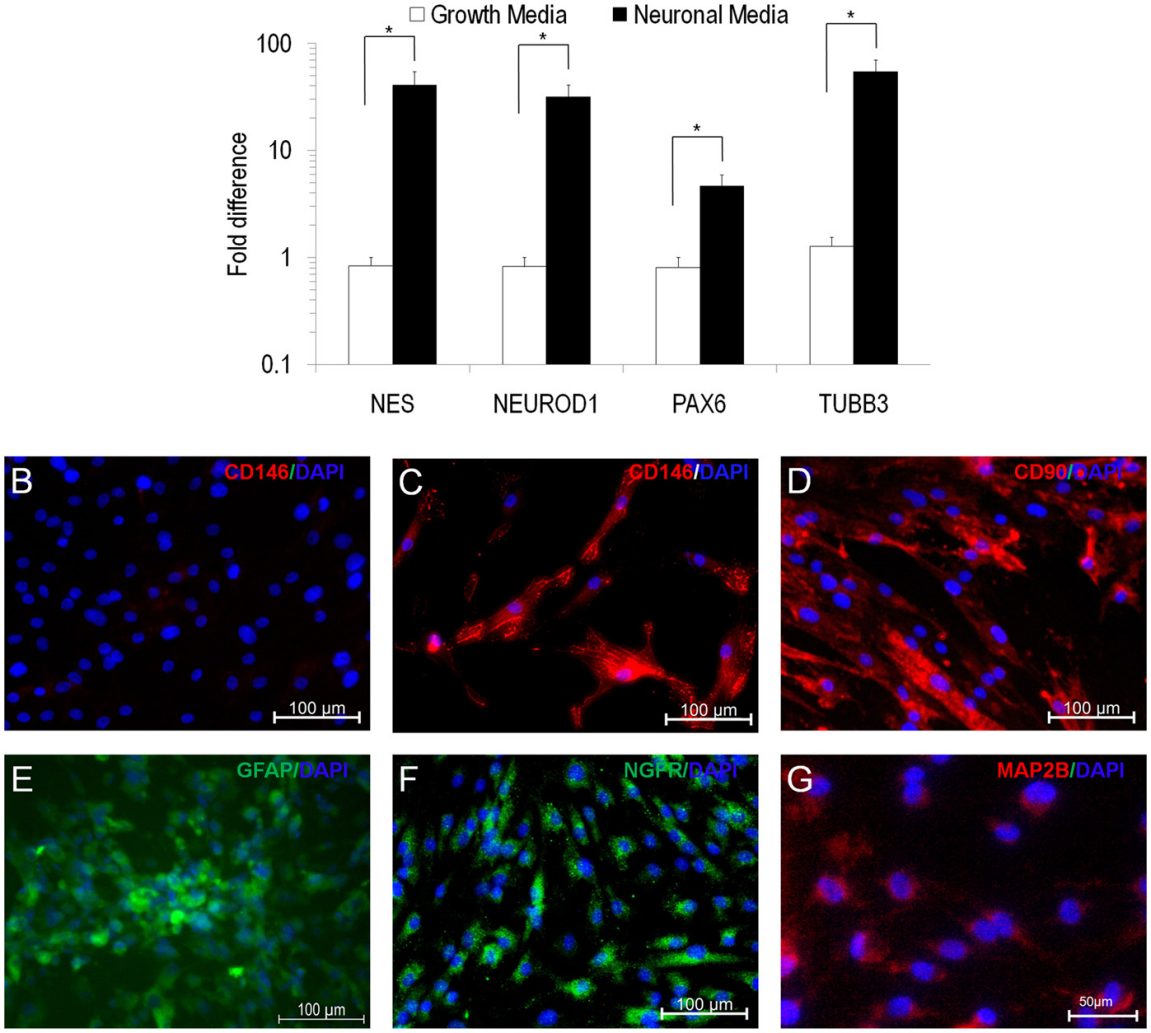
Neuronal differentiation of EOM-MSC. (A) Real-time PCR analysis of neuronal specific genes NES, NEUROD1, PAX6 and TUBB3 in EOM-MSC cultured in growth media and neuronal differentiation media for 14 days. The expression levels of the genes were normalized to GAPDH expression levels in the respective samples. Values are Mean±SEM, n = 5, *p<0.05. Immunoflurescence analysis of neuronal differentiated EOM-MSC showing downregulation of CD146 in cells cultured in (B) neuronal media compared to cells grown in (C) normal growth media. (D) CD90 expression was unaffected in EOM-MSC cultured in neuronal media. Neuronal differentiated cells exhibited upregulation of neuronal lineage markers (E) GFAP, (F) NGFR and (G) MAP2B. CD90, CD146 and GFAP staining were performed 9–10 days after the addition of neuronal induction media whereas NGFR and MAP2B staining were performed after 21 days of neuronal induction. Representative microscopic images are shown.

Although MSC from different organs share a similar phenotype, it has been proposed that their differentiation ability is influenced by the resident tissue [6] from which they were isolated. Although EOM is of mesodermal origin, it is a high innervated tissue [16], we tested to see if they posses neuroectodermal differentiation potential. EOM-MSC were subjected to neuronal differentiation through an intermediate neurosphere stage or direct conversion. EOM-MSC readily differentiated into neuronal cells, which was evident by the observed change in morphology and expression of neuronal lineage specific genes. A significant upregulation of mRNA for the neural markers Nestin (NES)and β-III-tubulin (TUBB3) was observed in the cells induced with the neuronal media (Fig 4A). Further, the mRNA for neuronal transcription factors Neuronal differentiation 1 (NEUROD1) and Paired box 6 (PAX6) were upregulated five-fold and 45-fold respectively in the neuronal differentiating cells (Fig 4A). However, the embryonic transcription factors OCT4, SOX2 and NANOG expression was downregulated during neuronal differentiation (S1 Fig). Neuronal differentiation in the cells was also accompanied by decrease in the expression of CD146 (Fig 4B) compared to proliferating control cells (Fig 4C) but no change in CD90 expression was observed (Fig 4D). The neuronal differentiation was further confirmed by increase in Glial fibrillary acidic protein (GFAP) in the cells at neurosphere stage and nerve growth factor receptor (NGFR) and microtubule-associated protein 2B (MAP2B) expression in the differentiated cells by immunocytochemistry analysis (Fig 4E–4G).

**Fig 5:**
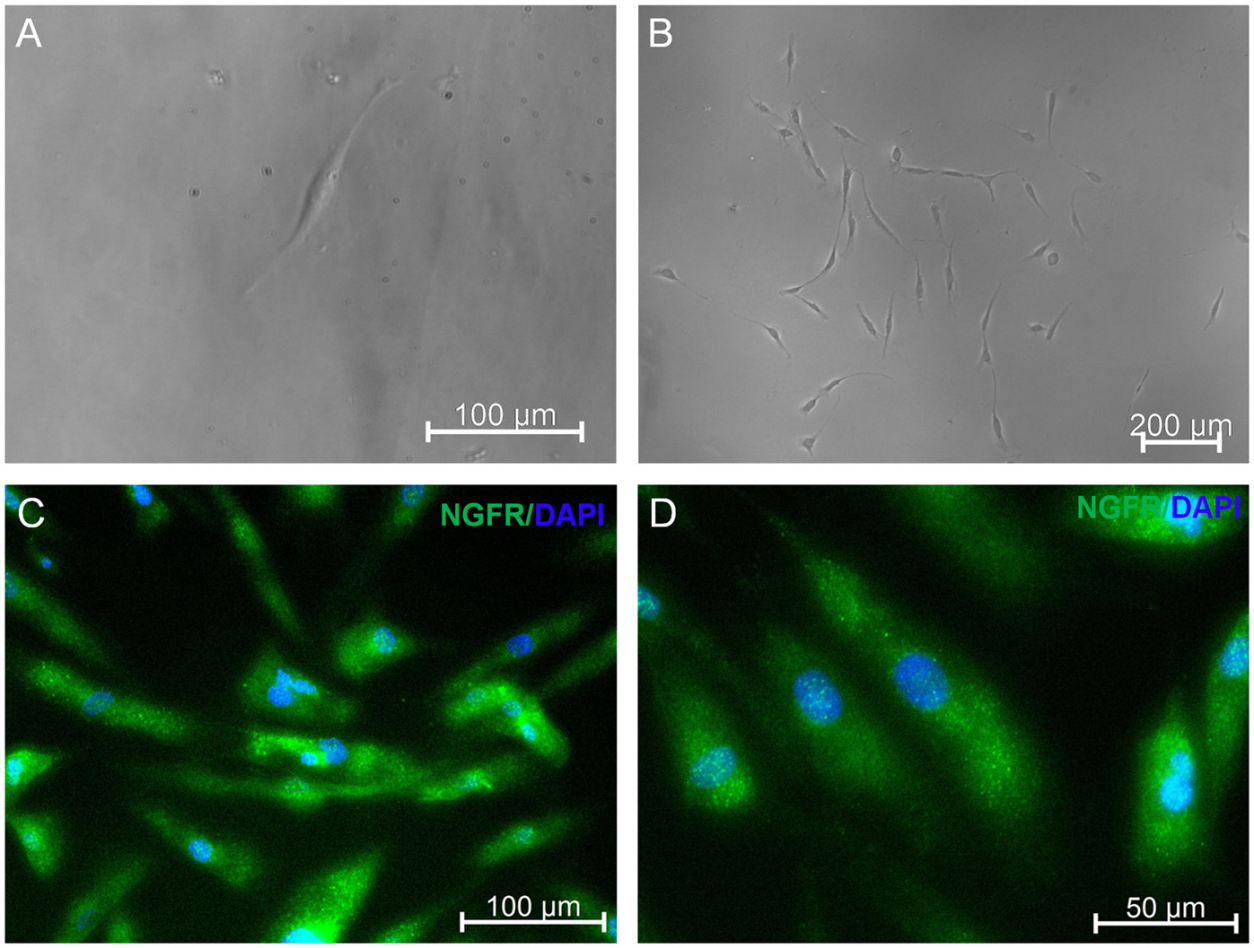
Neuronal differentiation of clonal derived EOM-MSC. (A) Microscopic image of single cell after 24hr of plating to isolate single cell derived colonies. (B) Single cell derived colony 7 days after plating. (C, D) Neuronal differentiation of single cell derived clones of EOM-MSC was determined by staining with NGFR 14 days after neuronal induction Representative microscopic images are shown.

To understand neuronal differentiation capacity of EOM-MSC further, single cell derived cell lines were obtained from EOM-MSC (Fig 5A and 5B). The clonally derived cells were expanded *in vitro* and could be differentiated into neuronal cells. Neuronal differentiation of clonal derived EOM-MSC was confirmed by expression of neural marker NGFR (Fig 5C and 5D).

### Gene Expression Analysis

**Fig 6:**
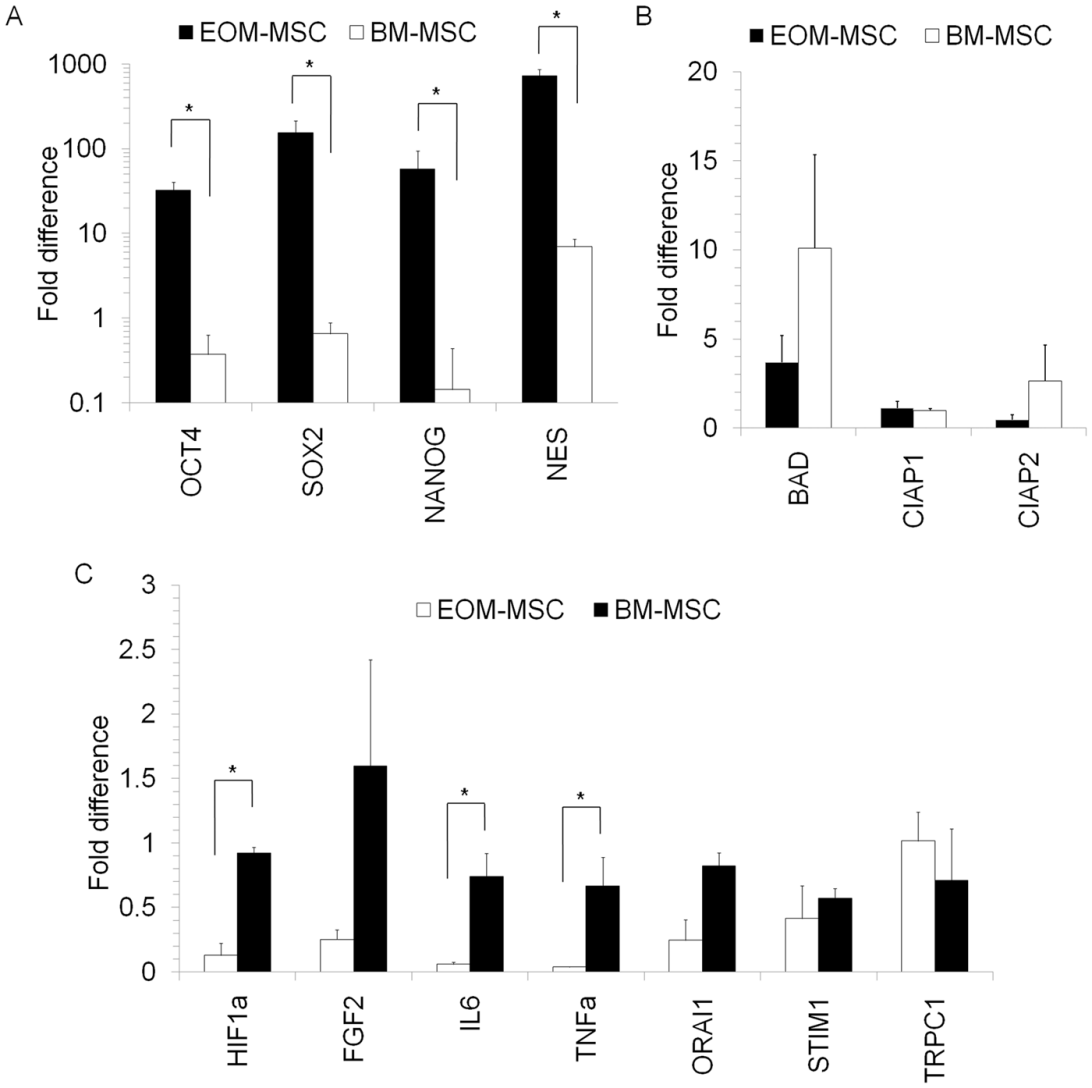
Gene expression analysis in EOM-MSC. (A) Expression levels of transcription factors OCT4, NANOG and SOX2 and neuronal lineage related gene NES in EOM-MSC was analysed by real-time PCR and compared with BM-MSC. mRNA expression levels of (B) apoptosis related genes BAD, cIAP1, cIAP2 and (C) other factors HIF1α, FGF2, IL6, TNFα as well as calcium channel related genes ORAI1, STIM1, TRPC1 were determined in EOM-MSC and BM-MSC by real-time PCR. The expression levels of the genes were normalized to GAPDH expression levels in the respective samples. Values are mean±SD, n = 3–5* p<0.05.

To study EOM-MSC further, we compared different groups of genes expressed in EOM-MSC with BM-MSC. Among the several genes anlaysed, mRNA expression of neuronal marker NES was significantly higher in proliferating EOM-MSC compared to BM-MSC (Fig 6A). In addition, mRNA of self-renewal related transcription factors OCT4, NANOG and SOX2 were also expressed60-100 fold higher in EOM-MSC thanin BM-MSC (Fig 6A). However, OCT4 protein expression was not detectable in the cells by immunocytochemistry analysis (S1 Fig). No significant difference in expression levels of apoptosis related genes BAD or inhibitor of apoptosis genes cIAP1 and cIAP2 was found between EOM-MSC and BM-MSC (Fig 6B). Factors such HIF1α, IL6 and TNFα were expressed higher in BM-MSC compared to EOM-MSC. Calcium channel related genes ORAI1, STIM1 and TRPC1 expression was similar in both EOM-MSC and BM-MSC (Fig 6C).

### Mitochondrial Distribution and Karyotype

Varum et al reported that the stem cells and differentiated cells exhibited varied mitochondrial distribution pattern inside the cells with stem cells characterized by peri-nuclear distribution, whereas the differentiated cells exhibited uniform cytoplasmic distribution [17]. The mitochondrial distribution in both EOM-MSC and BM-MSC were analyzed by staining with TMRE. A peri-nuclear mitochondrial distribution was observed in both EOM-MSC and BM-MSC (S2 Fig), however, cytoplasmic distribution was also seen in EOM-MSC suggesting a heterogeneous population of cells.

Lastly, to determine whether the cells underwent any chromosomal abnormality due to *ex vivo* expansion that will make these cells unsuitable for therapeutic purposes, karyotyping was performed to assess the chromosome number. All the cultures tested in different passages exhibited normal karyotype with 46 chromosomes without any gross abnormality (S3 Fig).

Thus, the adherent cells isolated from EOM tissues were morphologically and functionally comparable to MSC obtained from bone marrow and showed high neuronal differentiation capacity.

## Discussion and Conclusion

Several studies have enabled MSC to be used effectively for clinical applications. MSC have largely been shown to be isolated from bone marrow, adipose tissue and umbilical cord blood [7]. Each of these sources produced MSC which have the potential to differentiate into multiple cell types and MSC from ocular limbal stroma were also reported to exhibit multipotent differentiation capacity [18]. We show in our current study, extra ocular muscle (EOM) tissue as a novel tissue source of MSC with high neuronal differentiation capacity that could be used for both autologous and allogenic transplantations. Extra ocular muscles are important for controlling the movement of the eye but are excised and discarded during corrective strabismus surgery [19]and could be used for MSC isolation. Our study shows for the first time that multipotent mesenchymal stem cells are present in the human extra-ocular muscle tissues that could be successfully isolated and cultured without compromising their mesenchymal and non-mesenchymal differentiation ability. By merely plating the mechanically dissociated EOM tissue without any enzymatic treatment, spindle shaped cells migrated out of the tissue and formed adherent colonies with an average of 80% of samples yielding expandable colonies of cells. The cell surface expression of CD90, CD73, CD105, integrins and other markers along with spindle shaped morphology, mesenchymal differentiation capacity suggest that EOM-MSC resemble bone marrow or other mesenchymal tissue derived mesenchymal stem cells [20, 21]. Nevertheless, EOM-MSC that we isolated were unlike muscle progenitors as they were negative for CD34, a marker of muscle progenitor cells [22, 23]. EOM-MSC also did not resemble fibroadipogenic progenitors (FAP) reported by others as EOM-MSC were also negative for FAP marker PDGFRa [24–26].

EOM-MSC fulfills the criteria to be termed as MSC [27], and may be used in regenerative therapy for neurological disorders rather than bone repair as these cells seemed to have high neuronal differentiation capacity but low osteogenic differentiation compared to BM-MSC. Low osteogenic, adipogenic and chondrogenic differentiation ability of these cells could be related to the nature of tissue from which these cells were derived [28] which might be of advantage during cell therapy for neurological disorders. Similar to skin and adipose derived MSC, which have been proposed to be derived from mesoderm [29], EOM-MSC might also have a mesodermal origin. However, we found high expression of NESTIN in EOM-MSC which is not only a marker for neural progenitors but also expressed by neural crest cells [30, 31]. In addition, EOM-MSC expressed the cell surface marker SSEA4 which is expressed in high levels by embryonic stem cells. Although EOM-MSC showed higher expression levels of transcription factors OCT4, NANOG and SOX2 at mRNA level compared to BM-MSC, we could not identify OCT4 protein expression. Nevertheless, OCT4, NANOG and SOX2 transcript levels were downregulated during neuronal differentiation as reported also by others [32].

Taken together, we have identified previously unstudied multipotent stem cells from the extraocular muscle tissue that has the signature of mesenchymal stem cells with neuronal differentiation capacity *in vitro* which make them suitable candidates for use in treatment of neurological disorders.

## Supporting Information

S1 Fig **(A). OCT4 protein expression in EOM-MSC.** EOM-MSC were immunostained with anti-OCT4 antibody. Representative microscopic image is shown. **(B)OCT4, SOX2 and NANOG expressionduring neuronal differentiation.** Real-time PCR analysis of OCT4, SOX2 and NANOG in EOM-MSC cultured in growth media and neuronal differentiation media for 14 days. The expression levels of the genes were normalized to GAPDH expression levels in the respective samples. Values are Mean±SEM, n = 3–6.
(TIF)

S2 Fig Mitochondrial distribution. Mitrochoindrial distribution pattern in (A) EOM-MSC and (B) BM-MSC was determined by staining the cells in culture with TMRE. Representative images are shown.
(TIF)

S3 Fig Karyotype analysis of EOM-MSC. EOM-MSC showed normal number of chromosomes (passage 10–12).
(TIF)
