# Supplementary figures and images for: Mesenchymal Stem Cells from Human Extra Ocular Muscle Harbor Neuroectodermal Differentiation Potential
Source: PLoS One. 2016 Jun 1;11(6):e0156697. doi: 10.1371/journal.pone.0156697 (PMC4889147; doi:10.1371/journal.pone.0156697)

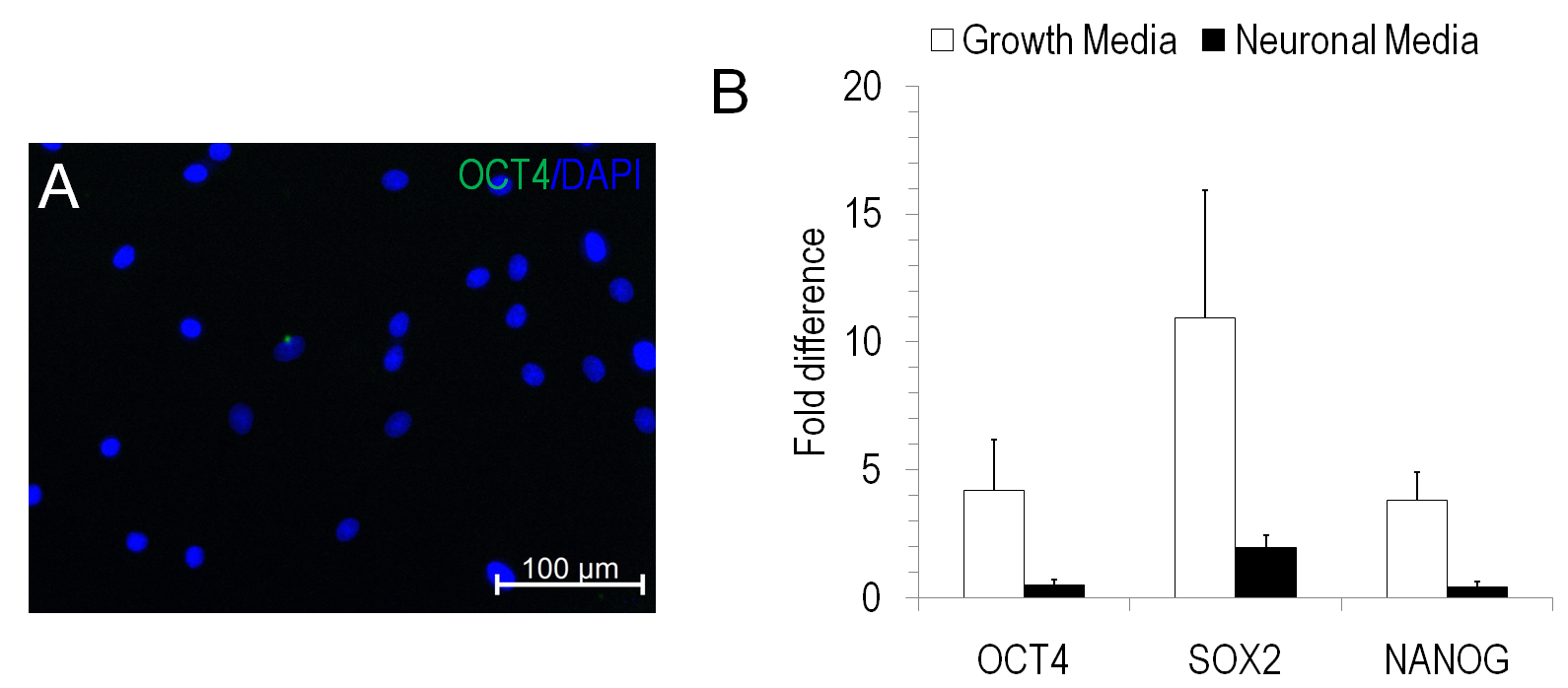

Supplement: S1 Fig — (A). OCT4 protein expression in EOM-MSC. EOM-MSC were immunostained with anti-OCT4 antibody. Representative microscopic image is shown. (B)OCT4, SOX2 and NANOG expressionduring neuronal differentiation. Real-time PCR analysis of OCT4, SOX2 and NANOG in EOM-MSC cultured in growth media and neuronal differentiation media for 14 days. The expression levels of the genes were normalized to GAPDH expression levels in the respective samples. Values are Mean±SEM, n = 3–6. (TIF) [file pone.0156697.s001.tif]

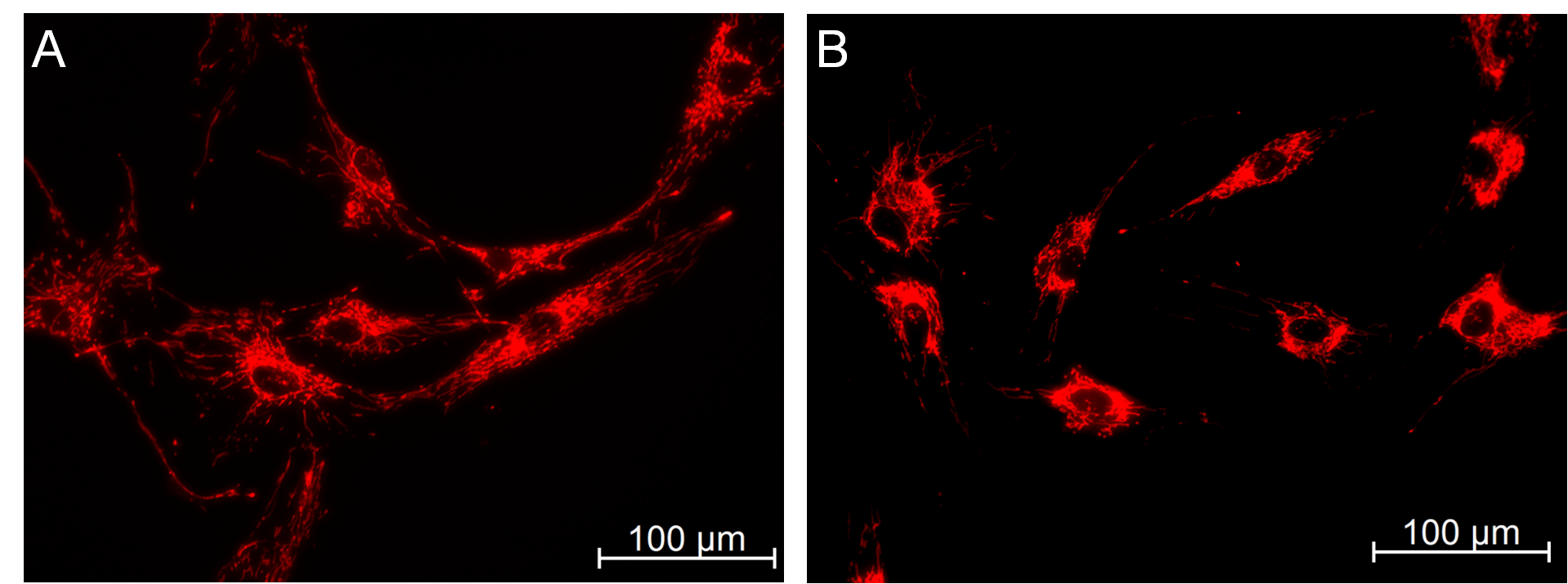

Supplement: S2 Fig — Mitrochoindrial distribution pattern in (A) EOM-MSC and (B) BM-MSC was determined by staining the cells in culture with TMRE. Representative images are shown. (TIF) [file pone.0156697.s002.tif]

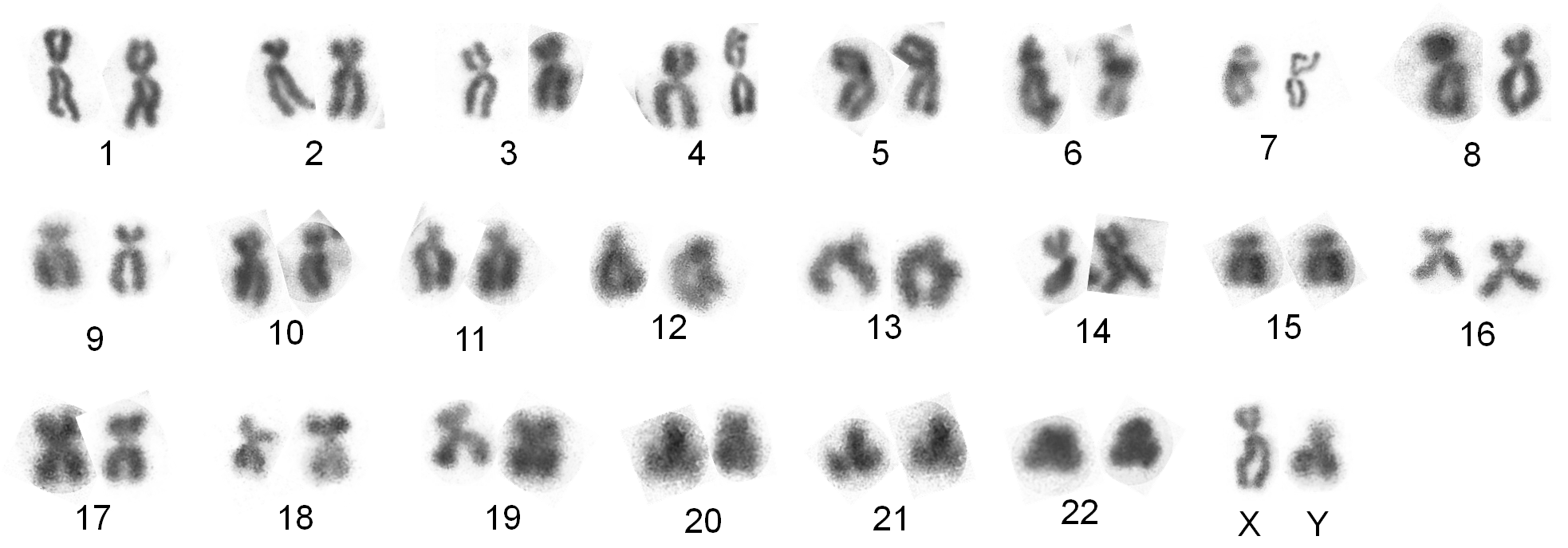

Supplement: S3 Fig — EOM-MSC showed normal number of chromosomes (passage 10–12). (TIF) [file pone.0156697.s003.tif]
